# Prevalence and impacts of poor sleep on quality of life and associated factors of good sleepers in a sample of older Chinese adults

**DOI:** 10.1186/1477-7525-10-72

**Published:** 2012-06-18

**Authors:** Catherine MH Lo, Paul H Lee

**Affiliations:** 1 RN BN Master of Gerontology Doctor of Health Sciences, School of Nursing, Faculty of Medicine, The University of Hong Kong, Hong Kong, People's Republic of China; 2 School of Public Health, Faculty of Medicine. The University of Hong Kong, Hong Kong, People's Republic of China

**Keywords:** Sleep, Older Chinese, Quality of life, Nursing

## Abstract

**Background:**

Sleep disturbance is a complex health problem in ageing global populations decreasing quality of life among many older people. Geographic, cultural, and ethnic differences in sleep patterns have been documented within and between Western and Asian populations. The aim of this study was to explore sleep problems among Hong Kong seniors by examining the prevalence of poor sleep quality, the relationship between sleep quality and health-related quality of life, and associated factors of good sleepers in different age groups.

**Methods:**

This cross-sectional study used convenience sampling and gathered data during face-to-face interviews. Older community-dwelling individuals (*n* = 301) were recruited in community centres in 2010. The Pittsburgh Sleep Quality Index and Medical Outcomes Study Short Form-36 were used to measure sleep quality and health-related quality of life. The Medical Outcomes Study Short Form-36 domain scores were compared between good and bad sleepers and between long and short sleepers using Hotelling’s T-Square test. SF-36 domain scores were placed into a logistic regression model that controlled for significant demographic variables (gender, educational level, perceived health).

**Results:**

Most (77.7%) participants were poor sleepers. Participants who had global Pittsburgh Sleep Quality Index scores <5 and slept ≥5.5 h/night had better health-related quality of life. Vitality, emotional role, physical functioning, and bodily pain domain scores were associated factors of good sleepers in different age groups.

**Conclusions:**

This study found a strong negative association between sleep deprivation (poor quality, short duration) and health-related quality of life. Associated factors for good sleep quality in later life differ among age groups in relation to universal age-related changes, and should be addressed by social policies and health-care programmes.

## Background

Within the context of global demographic ageing trends, health-care professionals and multidisciplinary researchers have recently begun to pay closer attention to sleep problems among the elderly. This attention has been the result of our increasing understanding of sleep and its relationship to health problems that lead to greater health-care utilisation. Sleep problems have been studied in Western countries for many decades, due to rapidly ageing European populations. Numerous studies [1,2] have indicated that up to 50% of European individuals aged 60+ years experience insomnia. In Asia, the ageing populations of Chinese societies (mainland China, Taiwan, and Hong Kong) have prompted research on sleep problems in older populations in the past two decades. Studies in Taiwan and mainland China [3-5] found that 6–38% of elderly individuals experienced insomnia. In Hong Kong, a previous study [6] examined sleep habits and disturbances among 1,034 elderly individuals, finding that 75% of these individuals had occasional or persistent sleep disturbance and 38% had insomnia. More recently, a study examined the prevalence of insomnia in the general population of Hong Kong among 5001 Chinese adults aged ≥18 years. It was found that the prevalence of insomnia was highest in the oldest age group (≥60 years; 41.7%) [7].

The aetiology of sleep disturbance in later life is multifactorial. Apart from the well-described age-related changes that result in poor sleep initiation, maintenance, and increased daytime napping in healthy ageing individuals [8], sleep disturbance in later life can also be associated with medical, psychological, social, demographic, and lifestyle factors [e.g. [6,9,10] . Previous studies have consistently associated poor sleep in later life with deprived quality of life (QOL). However, the causal relationships between poor sleep and medical conditions, such as depression, remain poorly defined [11,12]. Researchers have sought to identify the sociodemographic predictors of poor sleep in later life. Female gender, unmarried status, lower education and income levels, and advancing age were frequently found to be risk factors for poor sleep quality, shorter duration, sleep disturbance, and insomnia among older individuals [e.g. [4,13]. However, evidence for the extent to which these factors predict poor sleep has been heterogeneous.

Given the geographic, racial, and ethnic differences in sleep patterns and duration between Western and Asian populations, and among Chinese residents of different countries [14-18], sleep problems among older Chinese community-dwellers should be further explored. These differences may be due to the impact of different lifestyle choices on sleep quality, and the results of Western studies may not be generalisable to Asian populations. Rapid modernisation in Asian countries, such as Hong Kong, has also introduced environmental stressors that have further deprived individuals’ sleep quality [19]. Older Chinese individuals traditionally prefer to remain with their families and/or in their home communities, and thus rely on the support of community-care policies. Further research is vital to gain a better understanding of sleep problems in this population, and to guide the implementation of effective health-care programmes and policies that aim to improve sleep quality and QOL.

This paper is part of a study examining the effectiveness of an exercise programme to improve sleep quality among seniors in Hong Kong. The data in this study were extracted from baseline information collected before a 12-week exercise programme. Given the growing number of local and global older populations and increasing heterogeneity of the populations due to the increasing number of individuals over 80 years of age [20,21], we examined the prevalence of poor sleep quality and its relationship to health-related QOL (HRQOL) in a sample of older Chinese community-dwellers. We also explored the associated factors of good sleepers in different age groups.

## Method

### Design

This cross-sectional study employed a convenience sampling technique.

### Participants

Participants met the following eligibility criteria: (a) aged ≥60 years; (b) lived at home, rather than in an institution; (c) fully conscious, without cognitive impairment; (d) understood Cantonese; and (e) were willing to participate in a 12-week exercise programme.

Recruitment was conducted primarily in community centres and at centre-sponsored social activities in central and western Hong Kong. The first author and three trained research assistants recruited potential participants over a 4-month period in 2010. We approached elderly individuals attending social activities or seeking other services in the community centers. Once an individual expressed willingness to participate, his/her eligibility was assessed. Written consent was obtained, and a face-to-face interview was conducted in the community center or in a quiet nearby park location. A total of 318 eligible elderly were approached and 301 of them agreed to take part in the study. The response rate was nearly 97%.

### Data collection

#### Demographic variables

We used a structured questionnaire to record demographic variables, including age, gender, occupation, educational level, marital status, income, and living arrangement. Due to the small sample size, we dichotomised the following measures: perceived health: fair, good, or excellent/bad or very bad; marital status: single, divorced, or widowed/married; educational level: 0–6 years/7+ years; occupational status: full- or part-time/retired or unemployed; living arrangement: living alone/living with others; chronic illness: yes or no and financial status: sufficient/insufficient. Participants were divided into three age groups: 60–69 years, 70–79 years, and 80+ years. In this study, perceived health was defined as the overall health situation of the participants when comparing with individuals of their age. For financial status, the rank of sufficient referred to having adequate financial resources to maintain daily expense, and insufficient referred to not having adequate financial resources to maintain daily expense.

#### Sleep quality

The measurement of sleep quality includes quantitative (e.g. sleep duration, latency, number of arousals) and subjective (e.g. ‘depth’ or ‘restfulness’ of sleep) aspects [22]. We used the Pittsburgh Sleep Quality Index (PSQI), which has been employed previously to study sleep quality in older populations [4]. The 19-item PSQI includes seven components: subjective quality, latency, duration, efficiency, sleep disturbances, use of sleeping medication, and daytime dysfunction. Each component was scored (0–3) and the seven scores were summed to produce a global PSQI score (0–21); higher scores indicated poorer sleep quality [22].

This study used global PSQI and sleep-duration component scores to measure sleep quality. Poor sleepers were defined by global PSQI scores ≥ 5. Long sleepers were defined by a number of actual sleep hours per night equal to or larger than the median of sleep hours among all participants.

#### Health-related quality of life (HRQOL)

HRQOL was measured using the Chinese (Hong Kong) version of the Medical Outcomes Study Short Form-36 Survey (SF-36). This 36-item instrument is well-validated [23] and has been used widely in Hong Kong, including in older populations [24]. The SF-36 consists of eight domains: physical functioning (PF), role limitations due to physical health problems (RP), bodily pain (BP), general health perceptions (GH), vitality (VT), social functioning (SF), role limitations due to emotional problems (RE), and mental health (MH). The scores for each domain range from 0 to 100, with higher scores representing better health.

### Ethical considerations

Ethical approval for the original exercise study was obtained from the Institutional Review Board of the University of Hong Kong, and all the participants were required to provide written consents.

### Data analysis

Statistical analyses were conducted using SPSS software (ver. 18; SPSS Inc., Chicago, IL, USA). Global PSQI scores were used to categorise the 301 subjects as poor (global PSQI ≥ 5) or good (global PSQI < 5) sleepers. The demographic variables of these two groups were compared using chi-squared test and independent sample *t*-test. To examine the bivariate relationship between domains of quality of life and quality of sleep, the eight SF-36 domain scores were compared between good and poor sleepers and between long and short sleepers using Hotelling’s T-square test. To identify the significant correlates of sleeping quality from the eight SF-36 domains of both age-specific and across age groups, logistic regression using the backward variable-selection method with entrance and removal levels of 0.05 and 0.10 were conducted separately for each age group for the complete sample. Incidentally, the multicollinearity in SF-36 domains (inter-domain correlation ranged from 0.34 to 0.65) had been handled as domains with high correlation would be removed due to large p-value. Only variables that show significant association with good sleepers were included in the backward logistic regression model. To assess the predictive power of the above backward logistic regression models, classification rate, sensitivity, specificity, and area under the receiver operating characteristic curve (AUC) were computed. In this study, AUC lies between 0.5 and 1.0, and a value of 0.7 or above was considered to indicate a good prediction. We considered p < 0.05 to indicate statistical significance in all two-tailed tests. Results were presented as means ± standard deviations (SD).

### Validity and reliability of PSQI

The original English version PSQI was adopted in this study [22]. The first author translated the PSQI instrument from English into Chinese. A professional translator who was blinded to the original English version then performed a back-translation. The conceptual and content equivalence of the translated instrument were rated highly by a professional panel that included one bilingual psychologist and two nurse educators, indicating conceptual and content validity. A pilot test was conducted with 50 seniors to assess the readability and comprehension of the Chinese version of the PSQI. These individuals, who were subsequently included in the main study, had no difficulty understanding and completing the instrument. A 3-week test-retest reliability assessment was conducted with the same sample. Paired *t*-tests found no significant difference in the global PSQI or component scores of weeks 1 and 3. Pearson’s product–moment correlations demonstrated the stability of the global (r = 0.82; p < 0.001) and component (r = 0.45–0.82; p < 0.001) scores. Cronbach’s alpha coefficients for the seven component scores were 0.773 (week 1) and 0.751 (week 3). The first author and a research assistant conducted the pilot study and test-retest procedure. We did not perform inter-rater reliability as the content of the PSQI was simple and straight forward.

## Results

### Demographic characteristics

Most of the 301 participants were female and poor sleepers. The mean age of the sample was 76.08 ± 7.59 years (range: 60–96 years), and no significant difference in mean age was found between poor (76.06 ± 7.60 years) and good (76.15 ± 7.60 years) sleepers (difference = 0.09years, p = 0.93) or between long- (75.50 ± 7.49 years) and short-duration (76.82 ± 7.67 years) sleepers (difference = 1.32 years, p = 0.44). Study participants slept an average of 5.51 ± 1.5 h/night (range: 1.5–9.5 h/night). In addition, study participants who had received <7 years of education and/or perceived their health as poor or very poor were more likely to be poor and short-duration sleepers than their counterparts. The demographic characteristics and prevalence of poor sleep quality are shown in Table 1.

**Table 1:** Participant demographics by sleeping quality and sleeping duration

	**All participants**(*n* = 301)	**Good sleepers**	**Poor sleepers**	** *Test statistic/* **	**Long sleepers**	**Short sleepers**	** *Test statistic/* **
		**(*n* = 67)**	**(*n* = 234)**	** *P* **^** *a* **^	**(*n* = 170)**	**(*n* = 131)**	** *P* **^** *a* **^
**Age group**							
60–69 years	58 (19.3%)	14 (20.9%)	44 (18.8%)	*χ*^2^(2) = 0.17/	37 (21.8%)	21 (16.0%)	*χ*^2^(2) = 1.65/
70–79 years	135 (44.9%)	29 (43.3%)	106 (45.3%)	0.92	75 (44.1%)	60 (45.8%)	0.44
80+ years	108 (35.9%)	24 (35.8%)	84 (35.9%)		58 (34.1%)	50 (38.2%)	
**Gender**							
Male	47 (15.6%)	17 (25.4%)	30 (12.8%)	*χ*^2^(1) = 6.23/	31 (18.2%)	16 (12.2%)	*χ*^2^(1) = 2.04/
Female	254 (84.4%)	50 (74.6%)	204 (87.2%)	0.01	139 (81.8%)	204 (87.8%)	0.15
**Marital status**							
Single/divorced/widowed	197 (65.4%)	39 (58.2%)	158 (67.5%)	*χ*^2^(1) = 2.00/	106 (62.4%)	91 (69.5%)	*χ*^2^(1) = 1.66/
Married	104 (34.6%)	28 (41.8%)	76 (32.5%)	0.16	64 (37.6%)	40 (30.5%)	0.20
**Education level**							
0–6 years	228 (75.7%)	43 (64.2%)	185 (79.1%)	*χ*^2^(1) = 6.28/	119 (70.0%)	109 (83.2%)	*χ*^2^(1) = 7.02/
7+ years	73 (24.3%)	28 (35.8%)	49 (20.9%)	0.01	51 (30.0%)	22 (16.8%)	0.008
**Occupational status**							
Full-/part-time	7 (2.2%)	2 (2.9%)	5 (2%)	*χ*^2^(1) = 0.17/	3 (1.8%)	4 (3.1%)	*χ*^2^(1) = 0.54/
Retired/unemployed	294 (97.7%)	65 (97.0%)	229 (97.9%)	0.69	167 (98.2%)	127 (96.9%)	0.46
**Living arrangement**							
Live alone	112 (37.2%)	20 (29.9%)	92 (39.3%)	*χ*^2^(1) = 2.00/	59 (34.7%)	53 (40.5%)	*χ*^2^(1) = 1.05/
Live with others	189 (62.8%)	47 (70.1%)	142 (60.7%)	0.16	111 (65.3%)	78 (59.5%)	0.31
**Financial status**							
Sufficient	274 (91.0%)	64 (95.4%)	210 (89.7%)	*χ*^2^(1) = 2.13/	159 (93.5%)	115 (87.8%)	*χ*^2^(1) = 2.99/
Insufficient	27 (9.0%)	3 (4.5%)	24 (10.3%)	0.14	11 (6.5%)	16 (12.2%)	0.08
**Perceived health**							
Fair, good, or excellent	230 (76.4%)71 (23.6%)	60 (89.6%)7 (10.4%)	170 (72.6%)64 (27.4%)	*χ*^2^(1) = 8.26/ 0.004	140 (82.4%)30 (17.6%)	90 (68.7%)41 (23.6%)	*χ*^2^(1) = 7.65/ 0.006
Poor or very poor	71 (23.6%)	7 (10.4%)	64 (27.4%)	0.004	30 (17.6%)	41 (23.6%)	0.006
**Chronic illness**							
Yes	237 (78.7%)	50 (74.6%)	187 (79.9%)	*χ*^2^(1) = 0.87/	132 (77.6%)	105 (80.2%)	*χ*^2^(1) = 0.28/
No	64 (21.3%)	17 (25.4%)	47 (20.1%)	0.35	38 (22.4%)	26 (19.8%)	0.60
**Global PSQI score**					5.0 (3.25)	11.0 (4.0)	Z = 12.78/ ≤ 0.001
Median (IQR)	7.0 (5.5)						
Range	0-20						
**Sleep duration (hours per day)**							
Median (IQR)	5.5 (1.0)	7.0 (1.0)	5.0 (2.0)	Z = 9.39/ < 0.001			
Range	1.5-9.5						

PSQI: Pittsburg Sleep Quality Index.

^a^ Differences between good sleepers and poor sleepers.

Good sleeper: global PSQI score < 5. Poor sleeper: global PSQI score ≥5.

Long sleeper: sleep duration ** ≥ **5.5 hours per day. Short sleeper: sleep duration <5.5 hours per day.

### Sleep quality and health-related quality of life

Among the 301 participants, poor sleepers had significantly lower scores in all SF-36 domains (*F*_8,293_ = 1494.3, p < 0.001), with the largest difference in the BP domain (79.21 vs 60.76, p < 0.001) and the smallest difference in the PF domain (81.57 vs 74.29, p < 0.01). Domain rank orders were similar for good and poor sleepers, with the highest scores in the SF domain and lowest scores in the GH domain (Table 2).

**Table 2:** Comparison of the SF-36 derived Health-related Quality of Life domain scores of good and poor sleepers, and comparison among long sleepers (sleep duration ≥5.5 hours per day) and short sleepers (sleep duration <5.5 hours per day)

	**All participants**	**Good sleepers**	**Poor sleepers**	**Long sleepers**	**Short sleepers**
	**( *n* ** **= 301)**	**( *n* ** **= 67)**	** *n* ** **= 234)**	**( *n* ** **= 170)**	**( *n* ** **= 131)**
Physical Functioning	75.91 (17.62)	81.57** (15.13)	74.29 (17.97)	78.65** (15.86)	72.37 (19.16)
Role-Physical	78.76 (22.54)	84.98** (19.37)	76.97 (23.10)	81.98** (20.46)	74.57 (24.43)
Bodily Pain	64.86 (28.45)	79.21*** (22.90)	60.76 (28.59)	71.54*** (25.50)	56.20 (29.80)
General Health	50.24 (19.77)	58.27*** (19.04)	47.94 (19.40)	53.53** (18.68)	45.97 (20.38)
Vitality	61.09 (23.37)	72.57*** (18.49)	57.80 (23.61)	65.91** (21.23)	54.82 (24.58)
Social Functioning	88.08 (22.33)	94.96** (12.51)	86.11 (24.09)	91.25* (17.98)	83.97 (26.46)
Role-Emotional	83.31 (20.47)	89.55** (16.50)	81.52 (21.17)	85.39* (20.12)	80.60 (20.68)
Mental Health	74.25 (18.93)	83.36*** (13.91)	71.65 (19.39)	78.12*** (16.32)	69.24 (20.88)

Data are presented as mean (standard deviation).

*/**/*** Difference between good and poor sleeper significant at the 5%, 1%, and 0.1% level respectively (Hotelling’s T-square test on transformed value that maximizes multivariate normality).

Good sleeper: global PSQI score < 5. Poor sleeper: global PSQI score ≥5.

### Sleep duration and health-related quality of life

Among the 301 participants, long-duration sleepers (≥5.5 h/night) had higher scores than short-duration sleepers (<5.5 h/night) for all SF-36 domains (*F*_8,293_ = 2003.9, p < 0.001), with the largest difference in the BP domain (71.54 vs 56.20, p < 0.001) and the smallest difference in the RE domain (85.39 vs 80.60, p < 0.05). Domain rank orders were again similar for long and short sleepers, with the highest scores in the SF domain and lowest scores in the GH domain (Table 2).

### Correlates of good sleepers by age group

The incidence of good sleepers (predictive probability value > 0.3) was low (<30%). The logistic regression model including all participants showed that BP [odds ratio (OR) for 1-point increase: 1.019, 95% confidence interval (CI): 1.007–1.032, p = 0.002] and VT (OR for 1-point increase: 1.025, 95% CI: 1.009–1.041, p = 0.003) were significant correlates of good sleepers. A 10-point increase of BP and VT were both associated with 1.26 times the odds of being good sleepers. In age-group analyses, we found that VT (OR for 1-point increase: 1.041, 95% CI: 1.004–1.099, p = 0.034) and RE (OR for 1-point increase: 1.046, 95% CI: 0.995–1.099, p = 0.073) were correlates of good sleepers among participants aged 60–69 years. Among those aged 70–79 years, PF (OR for 1-point increase: 1.046, 95% CI: 1.000–1.097, p = 0.029) and VT (OR for 1-point increase: 1.037, 95% CI: 1.007–1.067, p = 0.033) were correlates of good sleepers, and BP (OR for 1-point increase: 1.033, 95% CI: 1.012–1.054, p = 0.002) was the only correlate of good sleepers among participants aged 80+ years (Table 3).

**Table 3:** Backward logistic regression models for prediction of good sleepers divided by age group, and their performance of prediction

	**All participants**	**Age 60-69**	**Age 70-79**	**Age 80+**
	**( *n* ** **= 301)**	**( *n* ** **= 58)**	**( *n* ** **= 135)**	**( *n* ** **= 108)**
Constant (Odds ratio)	0.015	0.0002	0.0008	0.029
Dependent variable	Bodily Pain	Vitality	Physical Functioning	Bodily Pain
(Odds ratio, 95% confidence interval)	(1.019** (1.007, 1.032))	(1.051* (1.004, 1.099))	(1.046* (1.000, 1.097))	(1.033** (1.012, 1.054))
	Vitality	Role-Emotional	Vitality	
	(1.025** (1.009, 1.041))	(1..046 (0.995, 1.099))	(1.037* (1.007, 1.067))	
				
Classification rate	0.71	0.74	0.73	0.69
Sensitivity	0.51	0.64	0.48	0.54
Specificity	0.77	0.77	0.79	0.74
AUC	0.72###	0.79##	0.77###	0.72##

Variables that show significant association with good sleepers were included in the backward logistic regression model.

The dependent variables were sorted according to ascending level of significance.

AUC: area under the receiver operating characteristic curve (≥0.7: good prediction).

*/**/*** significant at the 5%, 1%, and 0.1% level respectively.

#/##/### significantly different from 0.5 at the 5%, 1%, and 0.1% level respectively.

Dependent variable: good sleeper = 1; poor sleeper = 0.

The predictive power for all four logistic regression models was good, with classification rates ranging from 0.69 to 0.74 and AUC ranging from 0.72 to 0.79. The four models showed two- to three-fold greater sensitivity (range: 0.48 to 0.64; Table 3) than the random prediction of good sleepers (sensitivity: 0.22).

## Discussion

This study was limited by the over-representation of women and under-representation of men in the sample, likely due to our recruitment of participants at community centres (used more commonly by women). However, the results of this study can be applied in other Chinese cities with increasing numbers of older women, such as Shanghai with similar socio-economic contexts to that of Hong Kong [25,26].

The prevalence of poor sleep quality (78%) among Chinese seniors in our sample was similar to that found in a previous Hong Kong study [6], which used a more representative sample comprised of nearly equal numbers of older men and women. However, the prevalence of sleep problems in our sample was higher than found in many Western studies [e.g. [10]. This discrepancy may be due to the use of different sleep measurements, prohibiting direct comparison. The high proportion of poor sleepers may also reflect the demographic composition of our sample (84% women); more women than men have been found to experience sleep disturbances [27], whereas male gender has been associated positively with good sleep quality [13]. The prevalence of low educational levels and poor perceived health in our sample also increased the risk of poor sleep quality [3,4]. With respect to this, the effects of lifestyle factors on sleep problems among Hong Kong seniors require further investigation. In addition, the average sleep duration of our participants was lower than found previously among older adults in mainland China (7.5 h/night; [1]) and Spain (women: 7.9 h/night, men: 8.2 h/night; [28]) which is likely due to lifestyle differences among these populations and/or differences in the measurement of sleep duration.

In our sample, the HRQOL of poor and good sleepers, and that of long- and short-duration sleepers, differed significantly in all SF-36 domains (Tables 2). Because these groups did not differ in age, this difference did not reflect the age-related progression of physical frailty; it was more likely due to the negative impacts of poor sleep quality on physical and psychosocial aspects of QOL, such as pain [29]. Our literature review found that numerous studies had explored the relationship between sleep problems and HRQOL while the results were inconsistency. For instance, one population-based cohort study in Spain [28] found a similar relationship between short sleep duration and low HRQOL, another community-based study in Spain [30] found no such relationship. Such conflicting results also suggest that further research is necessary to clarify the relationship between sleep duration and QOL.

After controlling for demographic variables, we identified associated factors of good sleepers in different age groups. BP in advanced age has been identified as an independent risk factor for insomnia in Chinese and Western populations [e.g. [31], and lesser BP was associated factor of good sleepers in our 80 + −year age group. This result may reflect the greater frequency in advanced age of chronic medical conditions accompanied by severe BP (e.g. lower-back pain, osteoarthritis). Individuals of advanced age are also more likely to accept a reduction in energy due to physical frailty [32], which may have reduced the impact of VT on sleep quality. In contrast, the association of better VT, PF, and RE scores with good sleep quality in younger participants (60–79 years old) was likely due to a significant increase in physical problems and loss of social roles after retirement. These findings reflect the influence of universal, age-related, physical and psychosocial changes on sleep quality, and can facilitate the development of customised interventions for seniors of different ages in many cultures. For instance, social policies that support active ageing after retirement, such as the engagement of volunteers in research and community activities, can reduce social exclusion, increase vitality, and delay functional disabilities. Health-care interventions that promote culturally appropriate exercise programmes (e.g. tai chi, yoga) have been proven to improve VT and chronic BP and should be promoted among seniors of advanced age [33]. One of the potential limitations of the cross-sectional nature of this study is that the directions of the relationship between variables are unclear. Therefore, we cannot prove quality of life predicts sleep quality while we can only demonstrate there is a relationship between quality of life and sleep quality. Well-designed prospective studies to examine these effects are encouraged in the future.

## Conclusions

This study examined relationships between sleep quality and HRQOL among healthy Chinese seniors. Although this study was limited by the over-representation of women and under-representation of men and the cross-sectional design, our results provide strong evidence that sleep problems, including poor subjective quality and short duration, are negatively associated with HRQOL. The relationship we found between sleep duration and QOL is inconsistent with the findings of some previous studies. The associated factors of good sleepers differed in older and younger seniors; these differences reflect universal age-related progression rather than a culturally specific phenomenon, and can be used to enhance social policies and health-promotion programmes for older individuals in different cultures.

## Competing interests

The authors declare that they have no competing interests.

## Authors’ contributions

CL contributed to the design of the study, data collection and interpretation of the results. CL was also responsible for writing the manuscript. PL contributed to data analysis, interpretation of the results and manuscript preparation. All authors read and approved the final manuscript.

## References

[B1] Gureje O Kola L Ademola A Olley BO Profile, comorbidity and impact of insomnia in Ibadan study of aging International Journal of Geriatric Psychiatry 2009 24 7 686 93 10.1002/gps.2180 19156751 PMC2820713

[B2] LeBlanc M Merette C Savard J Ivers H Baillorgeon L Morin CM Incidence and risk factors of insomnia in a population-based sample Sleep 2009 32 8 1027 37 19725254 10.1093/sleep/32.8.1027 PMC2717193

[B3] Su T Huang S Chou P Prevalence and risk factors of insomnia in community dwelling Chinese elderly: a Taiwanese urban area survey Australian and New Zealand Journal of Psychiatry 2004 38 706 713 10.1080/j.1440-1614.2004.01444.x 15324335

[B4] Liu X Liu L Sleep habits and insomnia in a sample of elderly persons in China Sleep 2005 28 12 1579 1587 16408418

[B5] Xiang Y Ma X Cai Z Li S Xiang Y Guo H Hou Y Li Z Tao Y Dang W Wu X Deng J Lai K Ungvari G The prevalence of insomnia, its sociodemographic and clinical correlates, and treatment in rural and urban regions of Beijing, China: a general population-based survey Sleep 2008 31 12 1655 62 19090321 10.1093/sleep/31.12.1655 PMC2603488

[B6] Chiu H Leung T Lam L Wing YK Chung D Li SW Chi I Law WT Sleep problems in Chinese elderly in Hong Kong Sleep 1999 22 6 718 726 10.1093/sleep/22.6.717 10505817

[B7] Wong W & Fielding R: Prevalence of insomnia among Chinese adults in Hong Kong: a population-based study Journal of Sleep Research 2011 20 1 117 126 10.1111/j.1365-2869.2010.00822.x 20408932

[B8] Harrington JJ Lee-Chiong JJ Sleep and older patients Clinics in Chest Medicine 2007 28 4 637 84 10.1016/j.ccm.2007.07.002 17967287

[B9] Ersser S Wiles A Taylor H Wade S Walsh R Bentley T The sleep of older people in hospital and nursing homes Journal of Clinical Nursing 1999 8 4 360 8 10.1046/j.1365-2702.1999.00267.x 10624252

[B10] Morphy H Dunn KM Lewis M Boardman HF Croft PR Epidemiology of insomnia: a longitudinal study in a UK population Sleep 2007 30 3 274 280 17425223

[B11] Tsuruhei S Motoi I Haruo S Seiji M Takuji I Wakaba S Jun U Tsuyoshi M Isamu M Kyosuke K Ryutaro O Yoshiko S Rei Y Takumi M Takahiro M Soichi M Ken T Yasushi I Horiguchi J Sleep disturbances and depression in the elderly in Japan Psychiatry and Clinical Neurosciences 2003 57 265 270 10.1046/j.1440-1819.2003.01115.x 12753565

[B12] Fok M Stewart R Besset A Ritchie K Prince M Incidence and persistence of sleep complaints in a community older population International Journal of Geriatric Psychiatry 2010 25 37 45 19513987 10.1002/gps.2295

[B13] Gu D Sautter J Pipkin R Zeng Y Sociodemographic and health correlates of sleep quality and duration among very old Chinese Sleep 2010 33 5 601 610 20469802 10.1093/sleep/33.5.601 PMC2864875

[B14] Poland RE Rao U Lutchmansingh P McCracken JT Lesser RM Edwards C Ott GE Lin K REM sleep in depression is influenced by ethnicity Psychiatry Research 1999 88 95 105 10.1016/S0165-1781(99)00080-3 10622346

[B15] Jean-Louis G Magai CM Cohen CI Zizi F Gizycki H Dipalma J Casimir GJ Ethnic differences in self-reported sleep problems in older adults Sleep 2001 24 8 926 933 11766163 10.1093/sleep/24.8.926

[B16] Hale L Do P Racial differences in self-reports of sleep duration Sleep 2007 30 9 1096 1103 17910381 10.1093/sleep/30.9.1096 PMC1978399

[B17] Haseli-Mashhadi N Dadd T Pan A Yu Z Lin X Fransco OH Sleep quality in middle-aged and elderly Chinese: distribution, association factors and associations with cardio-metabolic risk factors BMC Public Health 2009 9 130 10.1186/1471/2458-9-130 19426521 PMC2683822

[B18] Xiang Y Ma X Lu J Cai Z Li S Xiang Y Guo H Hou Y Li Z Tao Y Dang W Wu X Deng J Lai K Ungvari G Relationships of sleep duration with sleep disturbances, basic socio-demographic factors, and BMI in Chinese people Sleep Medicine 2009 10 1085 1089 10.1016/j.sleep.2009.03.002 19442580

[B19] Stosic L Belojevic G Milutinovic S Effects of traffic noise on sleep in an urban population Arh Hig Rada Toksikol 2009 60 335 342 19789163 10.2478/10004-1254-60-2009-1962

[B20] United Nations World population ageing 2009 United Nations, New York

[B21] Census and Statistics Department Hong Kong Population Projection 2010–2039 The Government of Hong Kong Special Administrative, Region of the People’s Republic of China

[B22] Buysse DJ Reynolds CF Monk TH Berman SR Kupfer D J: The Pittsburg Sleep Quality Index: a new instrument for psychiatric practice and research Journal of Psychiatric Research 1989 28 2 193 213 10.1016/0165-1781(89)90047-4 2748771

[B23] Lam C Gandek B Ren XS Chan MS Tests of scaling assumptions and construct validity of the Chinese (HK) version of the SF-36 Health Survey Journal of Clinical Epidemiology 1998 51 11 1139 1147 10.1016/S0895-4356(98)00105-X 9817131

[B24] Woo J Lau E Lee P Kwok T Lau W Chan C Chiu P Li E Sham A Lam D Impact of osteoarthritis or quality of life in a Hong Kong Chinese population The Journal of Rheumatology 2004 31 12 2433 2438 15570647

[B25] Shangshai-Wikipedia, the free encyclopedia [http:en.wikipedia.org/wiki/Shanghai]7060577

[B26] HKCD [http://www.hkcd.com.hk/content/2011-05/06/content_2733563.htm]7060577

[B27] Li Y Wing YK Ho SC Fong SY Gender differences in insomnia: a study in Hong Kong Chinese population Journal of Psychosomatic Research 2002 53 601 609 10.1016/S0022-3999(02)00437-3 12127178

[B28] Faubel R Lopez-Garcia E Guallon-Castillor P Balboa-Castillo T Gutierrez-Fisac JL Bangegas JR Rodriguex-Artalego F Sleep duration and health-related quality of life among older adults: a population-based cohort in Spain Sleep 2009 32 8 1059 1068 19725257 PMC2717196

[B29] Chen Q Haymen L Sherling R Bean J Leveille S Characteristics of chronic pain associated with sleep difficulty in older adults: the maintenance of balance, independent living, intellect, and Zest in the elderly Boston study Journal of American Geriatric Society 2011 59 1385 1392 10.1111/j.1532-5415.2011.03544.x PMC3307096 21806564

[B30] Mesas AE Lopez-Gracia E Leon-Munoz M Luz M Graciani A Pilar G Fernando R The association between habitual sleep duration and sleep quality in older adults according to health status Age and Aging 2011 40 318 323 10.1093/ageing/afr004 21330338

[B31] Silwinski MM Sisto SA Gait, quality of life and their association following total hip arthroplasty Journal of Geriatric Physical Therapy 2006 29 1 8 15 10.1519/00139143-200604000-00003 16630370

[B32] Wadensten B Introducing older people to the theory of gerotranscendence Journal of Advanced Nursing 2005 52 4 381 388 10.1111/j.1365-2648.2005.03603.x 16268842

[B33] Williams K Abildso C Steinberg L Doyle E Epstein B Smith D Hobbs G Gross R Kelly G Cooper L Evaluation of the effectiveness and efficacy of Iyengar yoga therapy on chronic low back pain Spine 2009 34 19 2066 2076 10.1097/BRS.0b013e3181b315cc 19701112 PMC4393557

